# Supplementary material for: Resolving the full spectrum of human genome variation using Linked-Reads
Source: Genome Res. 2019 Apr;29(4):635–45. doi: 10.1101/gr.234443.118 (PMC6442396; doi:10.1101/gr.234443.118)
Supplement: Supplemental Material [file supp_gr.234443.118_Supplemental_longranger-2.2.zip › longranger-2.2.tar.bz2!/lr2.2-public/lib/stan/cmdstan/stan/doc/api/html/check__cholesky__factor_8hpp_source.html]

Stan: src/stan/math/error\_handling/matrix/check\_cholesky\_factor.hpp Source File


|  |  |
| --- | --- |
|  | Stan  2.5.0  probability, sampling & optimization |


- Main Page
- Related Pages
- Namespaces
- Classes
- Files

- File List
- File Members

All Classes Namespaces Files Functions Variables Typedefs Enumerations Enumerator Friends Macros Pages

- src
- stan
- math
- error\_handling
- matrix

check\_cholesky\_factor.hpp

Go to the documentation of this file.

1 #ifndef STAN\_\_MATH\_\_ERROR\_HANDLING\_\_MATRIX\_\_CHECK\_CHOLESKY\_FACTOR\_HPP

2 #define STAN\_\_MATH\_\_ERROR\_HANDLING\_\_MATRIX\_\_CHECK\_CHOLESKY\_FACTOR\_HPP

3

4 #include <stan/math/matrix/Eigen.hpp>

5 #include <stan/math/error\_handling/check\_positive.hpp>

6 #include <stan/math/error\_handling/check\_less\_or\_equal.hpp>

7 #include <stan/math/error\_handling/matrix/check\_lower\_triangular.hpp>

8

9

10 namespace stan {

11  namespace math {

12

29  template <typename T\_y, typename T\_result>

30  inline bool check\_cholesky\_factor(const char\* function,

31  const Eigen::Matrix<T\_y,Eigen::Dynamic,Eigen::Dynamic>& y,

32  const char\* name,

33  T\_result\* result) {

34  check\_less\_or\_equal(function,y.cols(),y.rows(),

35  "columns and rows of Cholesky factor",

36  result);

37  check\_positive(function, y.cols(), "columns of Cholesky factor",

38  result);

39  // FIXME: should report row i

40  check\_lower\_triangular(function, y, name, result);

41  for (int i = 0; i < y.cols(); ++i)

42  check\_positive(function, y(i,i), name, result);

43  return true;

44  }

45

46  }

47 }

48 #endif

check\_positive.hpp

check\_lower\_triangular.hpp

stan::math::check\_cholesky\_factor

bool check\_cholesky\_factor(const char \*function, const Eigen::Matrix< T\_y, Eigen::Dynamic, Eigen::Dynamic > &y, const char \*name, T\_result \*result)

Return true if the specified matrix is a valid Cholesky factor.

**Definition:** check\_cholesky\_factor.hpp:30

check\_less\_or\_equal.hpp

stan::math::check\_positive

bool check\_positive(const char \*function, const T\_y &y, const char \*name, T\_result \*result)

**Definition:** check\_positive.hpp:54

Eigen.hpp

stan::math::check\_less\_or\_equal

bool check\_less\_or\_equal(const char \*function, const T\_y &y, const T\_high &high, const char \*name, T\_result \*result)

**Definition:** check\_less\_or\_equal.hpp:52

stan::math::check\_lower\_triangular

bool check\_lower\_triangular(const char \*function, const Eigen::Matrix< T\_y, Eigen::Dynamic, Eigen::Dynamic > &y, const char \*name, T\_result \*result)

Return true if the specified matrix is lower triangular.

**Definition:** check\_lower\_triangular.hpp:27


---

[ Stan Home Page ]

*© 2011–2014,
Stan Development Team.*
